# Pharmacy in Mexico

**Published:** 1889-09

**Authors:** R. P. Daniel

**Affiliations:** Chihuahua, Mex.


### CULLINGS FROM CONTEMPORARIES.

From the Western Druggist.]

### PHARMACY IN MEXICO.

The requirements for practicing pharmacy in Mexico are of such a nature as to practically exclude foreigners, but for the entertainment of the readers of the Western Druggist, I will tell you what they are. The applicant must be a graduate, and his diploma will have to be accompanied by a certificate from the college stating that it is genuine; then there must be another certificate from the governor of the State in which the college is located, stating that the college is a reputable institution. This certificate has to be countersigned by the nearest Mexican minister, and should there be none in the State, it must be sent to Washington, D. C., and there signed by the Mexican minister, and from there it must be sent to the minister of foreign affairs at the city of Mexico for its final signature, and it is then recorded at the last named city.

After all of this "red tape" formality has been complied with the applicant is entitled to examination, which is all in Spanish. Having passed the examination satisfactorily he is paid a considerably smaller salary than similar talent would command in the United States.

As I am located about two miles from the city, I have had no good opportunity to notice the Mexican way of "running a drug store," and not being able to speak sufficient Spanish for conversational purposes I have made the acquaintance of but one druggist here, Mr. L. E. Lafon, who has the largest and nicest store in Chihuahua. He speaks good English, and I am indebted to him for a good deal of information in regard to the Mexican drug business. I am also under obligations to Dr. F. Paschal, chief surgeon of the Mexican Central railroad, for information regarding examination and registration of diploma. The same law is in force in regard to the practice of medicine.

Nearly all of the houses are built on the plan of a hollow square with a courtyard in the centre, a patio it is called, so the house is all around the yard, instead of vice versa. This makes the length of all houses parallel to the street, and only one counter is needed, which extends through the store from one end to the other. Excepting this the general arrangement of the stores is similar to those in the United States. Ointment jars are more numerous and make a very fine display, but I am told that most of them are empty and serve only as an ornament. Show globes are very popular, but the sign of the traditional mortar and pestle is absent from all of the stores except Mr. Lafon's. He is enterprising enough to keep quite a stock of American goods for his friends from the United States. The most common patent medicines are to be had here but at prices that seem surprising, but owing to the difference in value of Mexican and U. S. money, import duty, which is very high, and freight, too, is no small item of expense, so that $2.50 for Ayer's Sarsaparilla or 75 cents for Winslow's Soothing Syrup, is not as high as it seems, but at any rate it puts such luxuries (?) almost beyond the reach of poor people.

French and German chemicals and preparations are mostly used, as I am told that they are preferred on account of their greater purity. Prices and duty make all cost about the same, and if that is the only thing against American products it would seem that this is a fine field for the drug drummer to get in and do a little missionary work in persuading these people that we *can* make just as pure chemicals as the French or Germans.

The Pharmacopœia is not issued every ten years, nor at any regular interval. It is compiled by the various colleges of medicine in the City of Mexico, and is said to be largely, if not wholly, compiled from the French Codex.

The Mexicans have found out who owns the prescription, for it is the custom here to return it with the medicine. Such vague directions as "cucharadas" or "cucharapitas" are quite common. It means merely spoonfuls or teaspoonfuls; the old hackneyed "use as directed" is sometimes used, but "spoonful" seems to be the favorite. Of the six stores here only three have prescription cases, the others seem to get along without that necessary fixture. Three of the stores dispense soda water, but as they do not use enough ice and but very little foam it is not very good.

One of the most striking features of the city is the absence of awnings over the sidewalks, which are very narrow. The houses join each other and thus wall in the streets on both sides. There are but few show windows and none of them in the drug stores; and as all of the other windows have iron bars over them and closed with heavy shutters from within, every house looks like a prison. Show cases are not numerous enough to take up the greater part of the counter space, which may be according to the fancy of the proprietor, but is most likely because duty and freight are extremely high on that class of goods.

It is quite common for all stores to have names, and no drug store is complete without one. The following are specimens: "Botica Universal," "Botica Francesca," "Botica del Aguila," (The Eagle Drugstore) and in the City of Mexico there is one called "Almacen Central Beneficencia Publica," or the "central storehouse of public kindness." A drygoods store here goes by the name of "Grand Surprise."

The metric system is in most common use for prescriptions, though grains and drachms are still used by some, but only a few. The Spanish drachm has 73 grains, but as they are lighter than our grains it takes about 72 of them to equal 60 English grains. The pound (libra) is the standard of weight in commercial use for small quantities. The aroba (25 pds.) is used for larger quantities, and carga is used for still larger quantities. It is 300 pounds, and is considered a load for a "burro" (jackass.) The Spanish yard of 33 inches, called a vara, is used much oftener than the meter. The government has adopted the metric system and all duties are paid by the kilo, and railroad subsidies are granted at so much per kilometer. I will close this letter in the Mexican way by signing myself "S. S. S.," which has no reference to a popular patent medicine but means "your faithful servant," su suguro servidor.

CHIHUAHUA, MEX. R. P. DANIEL.

---

DR. M. L. HAGGARD has removed from Mountain Peak to Midlothian.

